# A Novel Antifungal System With Potential for Prolonged Delivery of Histatin 5 to Limit Growth of *Candida albicans*

**DOI:** 10.3389/fmicb.2019.01667

**Published:** 2019-07-30

**Authors:** Carolina R. Zambom, Fauller H. da Fonseca, Edson Crusca, Patrícia B. da Silva, Fernando R. Pavan, Marlus Chorilli, Saulo S. Garrido

**Affiliations:** ^1^Department of Biochemistry and Chemical Technology, Institute of Chemistry, UNESP – São Paulo State University, Araraquara, Brazil; ^2^Department of Drugs and Medicines, School of Pharmaceutical Sciences of Araraquara, UNESP – São Paulo State University, Araraquara, Brazil; ^3^Department of Biological Sciences, School of Pharmaceutical Sciences of Araraquara, UNESP – São Paulo State University, Araraquara, Brazil

**Keywords:** antifungal system, oral candidiasis, *Candida albicans*, Histatin 5, liposomes

## Abstract

Currently 75–88% of fungal infections are caused by *Candida* species, and *Candida albicans* is the main microorganism that causes these infections, especially oral candidiasis. An option for treatment involves the use of the antifungal peptide Histatin 5 (Hst 5), which is naturally found in human saliva but undergoes rapid degradation when present in the oral cavity, its site of action. For this reason, it is important to develop a way of applying this peptide to the oral lesions, which promotes the gradual release of the peptide. In the present study, we have evaluated the development of liposomes of different lipid compositions, loaded with the peptide as a way to promote its release slowly and gradually, preserving its antifungal potential. For this, the peptide 0WHistatin 5, an analog of the peptide Hst 5, was synthesized, which contains the amino acid tryptophan in its sequence. The solid phase synthesis method was used, followed by cleavage and purification. The liposomes were produced by thin film hydration technique in three different lipid compositions, F1, F2, and F3 and were submitted to an extrusion and sonication process to standardize the size and study the best technique for their production. The liposomes were characterized by dynamic light scattering, and tests were performed to determine the encapsulation efficiency, release kinetics, stability, and evaluation of antifungal activity. The extruded liposomes presented average size in the range of 100 nm, while sonicated liposomes presented a smaller size in the range of 80 nm. The encapsulation efficiency was higher for the sonicated liposomes, being 34.5% for F1. The sonicated F3 presented better stability when stored for 60 days at 4°C. The liposomes showed the ability to release the peptide for the total time of 96 h, with the first peak after 5 h, and a further increase of the released after 30 h. Time-kill assay showed that the liposomes were able to control yeast growth for 72 h. The data suggest that the liposomes loaded with 0WHistatin 5 maintained the action of the peptide and were able to limit the growth of *C. albicans*, being a suitable system for use in the treatment of oral candidiasis.

## Introduction

In recent decades, there has been an increase in mortality caused by fungal infections which have gone from isolated and rare cases to one of the greatest global public health problems especially among immunocompromised individuals (Robbins et al., 2016). This fact is related to medical interventions such as chemotherapy for cancer treatment, immunosuppression for transplantation, and the high prevalence of HIV infections (Berkow and Lockhart, 2017). This has allowed the appearance of prevalent fungal infections among which the main pathogens are *Candida albicans*, *Cryptococcus neoformans*, and *Aspergillus fumigatus* (Robbins et al., 2016). These pathogens cause the death of more than 1 million people annually in the world with 75–88% of fungal infections being caused by *Candida* species, which generated a cost of $ 1.7 billion for public health in the US and the increase of severe cases of hospital infections (Berkow and Lockhart, 2017).

*C. albicans* exists as a commensal microorganism of the skin, mouth, and gastrointestinal tract. Its spread is controlled by coexistence with the normal human microbiota and also by the processes of defense related to immune system. However, when there is suppression of the immune response against *C. albicans*, colonization of the tissue occurs establishing the infection, more commonly manifested as candidiasis and oropharyngeal candidiasis (Robbins et al., 2016).

Because it is a polymorphic microorganism, *C. albicans* is able to transition between the yeast and hypha lifestyles. The hypha has greater resistance and greater invasive capacity in tissues compared to the yeast. For this reason, this species demonstrates resistance to the most common antifungals, such as polyenes (nystatin and amphotericin B) or azoles (itraconazole, miconazole, and fluconazole) (Hawser and Douglas, 1995).

An alternative treatment is the use of fluconazole and amphotericin B, which are more effective but have higher levels of toxicity and should not be used in constant or routine doses (Johnson et al., 1995; Cannon et al., 2007). For this reason, the search for new treatment options and new drugs are constantly developing mainly in the biotechnology field. An example of this is the biologically active peptides naturally found in living organisms such as the antimicrobial peptides (AMPs) of Histatin class.

One of these AMPs, that is, the focus of this data is Histatin 5 (Hst 5), one of the peptides of the Histatin class naturally present in human saliva and potentially active against pathogenic yeast such as *C. albicans* (Helmerhorst et al., 1999; Baev et al., 2002). Hst 5 is a peptide of about 3 kDa composed of a linear sequence of 24 amino acid residues, has a positive charge at physiological pH, assumes α-helix structure in DMSO (dimethylsulfoxide) and TFE (trifluorethanol)/water, and in water preferably takes on a random structure (Seo et al., 2012).

Recent studies have demonstrated that Hst 5 is able to inhibit *C. albicans* growth in concentrations ranging from 25 to 800 μg ml^−1^ (Moffa et al., 2015b) with MIC of 25 μg ml^−1^ (Konopka et al., 2010). Another feature of this peptide is its ability to protect the oral epithelium from *C. albicans* infection, proven in *in vitro* studies with gingival fibroblast cells, in which at a concentration of 50 μg ml^−1^, there was no invasion of the cells by the microorganism (Moffa et al., 2015b).

However, we have reported that Hst 5 undergoes rapid degradation, and the proteolysis has been the major focus aimed at explaining that degradation causes the reduction or even loss of its antifungal activity (Moffa et al., 2015a). Another problem observed was that Hst 5 can interact with other proteins present in saliva, such as amylase, resulting in a complex free of antifungal activity (Moffa et al., 2015a).

One way to overcome this problem would be the use of nanocarriers, such as liposomes, that can incorporate the peptide and increase its availability at the site of action. Liposomes have been used in therapeutics for more than 40 years, presenting many advantages, including biocompatibility, because they are constituted by phospholipids, such as the biological membranes of the cells (Chorilli et al., 2013).

The liposome advantage is related to a longer duration of the therapeutic effect of the drug (De Araújo et al., 2003), prolonging its action and allowing larger doses to be administered without the risks of toxicity (Frézard et al., 2005). Thus, there are a lower number of drug administrations throughout the treatment, as demonstrated by studies with liposome-encapsulated LL-37 peptide which showed improved bioactivity and reduced toxicity for treatment of HSV-1. The studies carried out indicated the LL-37 liposomal formulation as an effective system for carrying and delivering the peptide to the action target (Ron-Doitch et al., 2016).

Liposomes are also effective in promoting protection against external degradation by enzymes or by proteolytic degradation (Voltan et al., 2016). For this reason, they are used for encapsulation of peptides and proteins. Liposomes encapsulated with peptide ghrelin, used to treat cachexia, characterized as extreme weakness in patients afflicted with chronic diseases, showed that liposomes were able to protect the peptide from the attack of trypsin and carboxylesterase enzymes by 20 and 81%, respectively (Salade et al., 2017).

Thus, these positive aspects about the use of liposomes motivated us to elaborate a system to apply Histatin 5, with the main interest in increasing peptide availability at the site of action, causing its fungicidal effect to be preserved and intensified over a longer period, thus optimizing treatment against *C. albicans*.

## Materials and Methods

### Chemicals and Microorganisms

Reagents N-α-fluorenylmethyloxycarbonyl-amino acids (Fmoc-amino acid) and Fmoc-tyr-wang resin were purchased from Novabiochem^®^. N-hydroxybenzotriazole (HOBt), N,N′-diisopropylcarbodiimide (DIC), dichloromethane (DCM), dimethylformamide (DMF), and trifluoroacetic acid (TFA) were purchased from Fluka^®^. Ethanedithiol (EDT), glacial acetic acid, dimethylformamide (DMF), dimethyl sulfoxide (DMSO), chloroform, and grade HPLC acetonitrile (ACN) were purchased from Merck^®^. The microorganism *C. albicans* (ATCC 90028) was donated by the National Institute of Quality Control in Health (INCQS – Fundação Oswaldo Cruz Brazil). All lipids were purchased from Sigma^®^.

### Peptide Synthesis

The peptide was synthesized manually according to the Fmoc chemistry (Merrifield, 1963). In each synthetic cycle, the deprotection of the α-amino group deprotection was performed with 20% piperidine in DMF for 20 min. The coupling reactions were performed with a threefold excess of DIC component and HoBt in DMF/DCM (1:1, v:v). After approximately 2 h of coupling, the ninhydrin test was performed to confirm the occurrence of the reaction. Final cleavage of the peptide from the resins and the deprotection of the side-chain protector groups were done by treatment with a solution containing TFA (94.5%), deionized water (2.5%), EDT (2.5%), and TIS (0.5%) at 25°C for 3 h. After the cleavage procedure, the crude peptides were precipitated with ethyl ether, separated from the soluble non-peptidic contents by centrifugation, extracted into 10% acetic acid in water and lyophilized.

The analog peptide 0WHistatin 5 (0WHst 5) was synthesized with the addition of the amino acid tryptophan (W) in amino terminal extremity, as can be seen in Table 1. Because it is a fluorescent amino acid, with excitation at the wavelength of 280 nm and emission of fluorescence at the wavelength of 360 nm, its addition allowed its monitoring by fluorescence technique, with greater sensitivity as can be seen in the following sections.

**Table 1:** Amino acid sequence of Histatin 5 and the synthesized 0WHistatin 5 analog peptide.

Peptide	Amino acid sequence
Histatin 5	D S H A K R H H G Y K R K F H E K H H S H R G Y
0WHistatin 5	**W** D S H A K R H H G Y K R K F H E K H H S H R G Y

The purification process of the crude peptides was performed in semi-preparative HPLC with a Zorbax Eclipse XDB C18 reverse phase column (9.4 mm × 250 mm and 5 μm). The qualitative analysis was performed analytically using a Shimadzu LC-10A/C-47A separation system coupled to a Shimadzu LC-10A/C-47A UV/Vis detector with a Waters Symmetry C18 column (2.1 mm × 150 mm and 5 μm). The chromatographic conditions in the semi-preparative mode were: solvents A (0.045% TFA.H_2_O) and B (0.036% TFA.ACN), gradient of 0.33%/min solvent B over 90 min, flow rate of 5 ml/min, and detection wavelength at 220 nm. For the analytical mode, the conditions were: solvents A (0.045% TFA.H_2_O) and B (0.036% TFA.ACN), gradient from 5 to 95% solvent B in 30 min, flow rate of 0.6 ml/min, and detection wavelength at 220 nm. After the purification procedure of the peptide, the molecule characterization was done using mass spectrometry. The analysis of pure peptides was performed by HPLC coupled to mass spectrometer operated in electrospray positive mode (LC/ESI-MS+) in a Bruker^®^ type Ion Trap Amazon SL mass spectrometer. This procedure allowed to determine the quality and identity of the sample simultaneously.

### Preparation of Liposomes

Three different liposome formulations were produced by the thin film hydration technique. The lipid film was composed of soy dipalmitoyl phosphatidylcholine (DPPC), cholesterol (Chol), polyethylene glycol (PEG), and 1-palmitoyl-2-oleoyl-sn-glycero-3-phospho-rac-1-glycerol (POPG). The lipid mixtures were dissolved in chloroform and then were evaporated under nitrogen flow, to form a thin lipid film in tube wall. For resuspension of the lipid film, solutions with or without the peptides solubilized in a 10 mM Tris HCl buffer, pH 7.4, was used. A suspension of large multilamellar vesicles (MLV’s) was obtained and submitted to two different techniques for homogenization: extrusion, using Avanti Polar Lipids^®^ extruder equipped with Nuclepore^®^ polycarbonate membrane, GE Healthcare Life Science, with pores of 100 nm, and sonication, using titanium tip sonicator (QSonica^®^ Q700).

### Physical Characterization of Liposomes

#### Determination of the Mean Hydrodynamic Diameter, Polydispersity Index, and Zeta Potential

The mean hydrodynamic diameter (Z-Ave or d.nm) and polydispersity index (PDI) were determined by dynamic light scattering (DLS, Zetasizer Nano NS, Malvern Instruments^®^, Malvern, UK). The zeta potential (ZP) of the liposomes was evaluated by the electrophoretic mobility of the particles according to the Helmholtz-Smoluchowski equation and processed using the Zetasizer Nano NS equipment software (Malvern Instruments). The suspensions of empty and loaded liposomes were diluted in 10 mM Tris HCl buffer, pH 7.4, in a 1:10 ratio. All experiments were run in triplicate at 25°C.

### Encapsulation Efficiency

The unencapsulated peptide separation was performed by molecular exclusion chromatography technique, using a Sephadex^®^ G-50 column, with 10 mM Tris HCl buffer, 150 mM NaCl, pH 7.4.

The fractions with liposomes were frozen in liquid nitrogen and lyophilized for 48 h. Then 3 ml of methanol were added to the samples, an aliquot of this solution was diluted in methanol and analyzed in spectrofluorometer to quantify the encapsulated peptide. Once the encapsulated peptide concentration was determined, the encapsulation efficiency, EE, was calculated using Equation 1.

$$\mathrm{EE}\;\left(\%\right)\;\;=\;\;\frac{\mathrm{Encapsulated peptide}}{\mathrm{Total added peptide}}\;\;\times \;\;100$$ (1)

### Formulation Stability

The produced formulations, F1, F2, and F3, were stored at 8°C and at 37°C. The mean size, polydispersity index, and zeta potential were analyzed after 1, 5, 10, 15, 30, 45, and 60 days of storage.

### *In vitro* Release Study

The release kinetics of the encapsulated peptide was performed using the fluorescence technique, since the inserted tryptophan (W) provides fluorescence to the molecule. Spectrofluorometer Varian^®^ CaryEclipse coupled to a circulation system of the solution 10 mM Tris HCl, pH 7.4, was used. About 1 ml of the solution of liposome and 0WHst 5 was added to a cellulose acetate dialysis membrane (Sigma-Aldrich^®^ cut-off 14,000 kDa).

The peptide that permeated the dialysis tube and exited into the buffer solution was monitored by the Kinetics Varian^®^ CaryEclipse software, with maximum fluorescence emission at 360 nm for 96 h. The maximum fluorescence was plotted against the time and, by the use of the standard curve, the total peptide released by the system was evaluated by the plot of the maximum released concentration against the time.

### Time-Kill Curve Studies

For the preparation of the standardized suspensions of *C. albicans* (ATCC 90028), an inoculum size of 1.10^5^ CFU ml^−1^ was used. Nine milliliters of Sabouraud Dextrose Broth (SDB) prepared with the microorganism were added in an Erlenmeyer for the growth control, 512 μg ml^−1^ of fluconazole was used for the positive control (cell death) and for testing the systems produced 1 ml of the liposomal system encapsulated or not with 0WHst 5 was added. The Erlenmeyers were then incubated at 37°C, under constant stirring, and aliquots of 100 μl were withdrawn and serially diluted every 3 h for 12 h. After this period, the aliquots were withdrawn and serial diluted every 12 h for 60 h. About 100 μl of each dilution were transferred to a plate with SDA and incubated for 48 h. The colonies in each plate were then counted.

### Statistical Analysis

*t*-Student test was used to verify the statistical difference between the last points of the time kill test curves. The level of significance was 90%. The growth control and fluconazole curves were performed in duplicate.

## Results and Discussion

### Preparation of Liposomes

In this work, three liposomal formulations were used according to the amounts shown in Table 2. The amounts are represented in mass. The peptide used was successfully obtained with high purity (>95%) and loaded into the liposomes as described in the methodology section.

**Table 2:** Lipid composition of the liposomes.

Formulation	DPPC	Chol	PEG	POPG
F1	40	2	–	–
F2	40	2	1	–
F3	40	2	–	1

**Figure 1:**
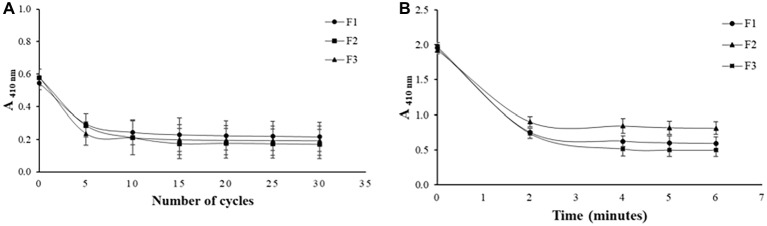
Effect of the amount of extrusion cycles on different liposome compositions **(A)** and effect of sonication time on different liposome compositions **(B)**.

To determine the amount of extrusion cycles and sonication time required for the formation of the liposomes, periodic measurements were made in a spectrophotometer (Chorilli et al., 2013). The results shown in Figure 1 indicates that there is a decrease in absorbance at 410 nm as a function of sonication time and amount of extrusion cycles, respectively, for all formulations developed.

All the formulations produced required 15 extrusion cycles to be formed, thus, the addition of PEG in F2 and POPG in F3 did not promote differences in the number of extrusion cycles observed for F1 which does not have any of these components. When PEG and POPG were added to the formulation of these liposomes, the main objective was to promote better features like fluidity and stability to vesicles. For the sonication technique, monitoring was performed after every 2 min, as seen in Figure 1B, and the stabilization of the absorbance values was achieved in the time of 4 min for all formulations. As in the extrusion, the addition of PEG and POPG in F2 and F3 did not affect the minimum time of 2 min of sonication observed for formulation F1 that does not contain these components.

### Physical Characterization of Liposomal Formulation

The mean size, zeta potential, and polydispersity index (PDI) for empty and loaded liposomes with 0WHst 5, can be seen in Tables 3 and 4.

**Table 3:** Size, PDI, and zeta potential for empty and loaded F1, F2, and F3 liposomes, obtained by extrusion.

Formulation	Size (nm)	PDI	Zeta potential (mV)
F1	106.1 ± 1.70	0.128 ± 0.06	−49.8 ± 1.17
F1/0WHst 5	116.6 ± 0.30	0.081 ± 0.10	−43.0 ± 0.50
F2	97.1 ± 1.77	0.099 ± 0.04	−38.5 ± 2.29
F2/0WHst 5	119.2 ± 0.80	0.087 ± 0.08	−42.2 ± 0.90
F3	105.1 ± 0.70	0.113 ± 0.06	−42.7 ± 1.89
F3/0WHst 5	112.6 ± 0.50	0.068 ± 0.08	−47.4 ± 0.50

**Table 4:** Size, PDI, and zeta potential for empty and loaded F1, F2, and F3 liposomes, obtained by sonication.

Formulation	Size (nm)	PDI	Zeta potential (mV)
F1	88.1 ± 8.03	0.371 ± 0.01	−51.4 ± 8.94
F1/0WHst 5	144.3 ± 0.35	0.255 ± 0.01	−48.6 ± 0.21
F2	96.4 ± 10.41	0.382 ± 0.05	−56.4 ± 2.28
F2/0WHst 5	147.4 ± 1.45	0.124 ± 0.09	−51.5 ± 1.79
F3	82.2 ± 4.77	0.287 ± 0.02	−56.5 ± 3.78
F3/0WHst 5	133.6 ± 0.82	0.241 ± 0.01	−52.3 ± 8.17

The mean size of the empty and sonicated liposomes was lower than for the empty liposomes obtained by extrusion. This can be observed by comparing the sizes for the empty liposomes obtained by extrusion and sonication seen in Tables 3 and 4.

This fact is related to the technique of obtaining the liposomes. The 100 nm pores of the polycarbonate membrane used in the extruder, ensures final sizes closer to 100 nm. In sonication, there is no way to control or predict the final mean size of the liposomes, which may vary as the amplitude, sonication time, and potency of the equipment change.

There was an increase in the mean size of loaded liposomes when compared to empty liposomes and this was observed for all formulations obtained by both techniques. According to Table 3, the empty F2 size was 97.2 nm, and after the addition of 0WHst 5, it had a size of 119.2 nm. For sonicated F3, the mean size before loading of the peptide was 82.2 nm, rising to 133.6 nm with 0WHst 5. The same can be observed sonicated F1, which after addition of 0WHst 5 presented a 144.3 nm, against 88.1 nm when empty. We observed an increase in size for liposomes after peptides had been encapsulated, and this indicates that 0WHst 5 was incorporated into the liposomes, probably in the inner aqueous compartment because it is a water-soluble molecule (Nii and Ishii, 2005; Mohan et al., 2016). These data are interesting from the point of view of the intended application, which aims to stabilize the peptide and promote a better availability of it at the site of action.

The zeta potential of the produced liposomes is negative for all formulations prepared. The zeta potential value became less negative for all sonicated formulations after incorporation of the peptide. 0WHst 5 is a positively charged peptide (net charge at pH 7: +5.7) that interferes with the final charge of the liposomes, making them less negative. The POPG lipid used in the F3 formulation is anionic and confers a greater amount of negative charges to the external lipid bilayer which may be less influenced by the positive charge of 0WHst 5. The standard zeta potential value used for this is ±30 mV (Ebrahimi et al., 2015; Berbel Manaia et al., 2017; Sato et al., 2017). The sonicated and extruded encapsulated F1, F2, and F3 formulations have zeta potential in the range of −30 to −50 mV, indicating that all formulations are stable.

PDI values in the range of 0.07 to 0.09 were found for all extruded formulations containing 0WHst 5, as can be seen in Table 3. Thus, it can be stated that there is a monodispersion. For sonication, PDI values of 0.371, 0.382, and 0.287 were found for empty F1, F2, and F3, respectively, indicating homogeneity of the analyzed sample. However, there are vesicles in other size ranges, characterizing a polydispersity.

The PDI values fell to 0.255, 0.124, and 0.241, respectively, when 0WHst 5 was loaded into the liposomes prepared by sonication. Addition of the peptide to the formulations made them more homogeneous. The same effect was also observed for the liposomes obtained by extrusion, with 0.128 of PDI for empty and 0.081 for loaded F1 formulation.

### Encapsulation Efficiency

The encapsulation efficiency (EE) for the 0WHst 5 is below 40%, as seen in Table 5. Addition of cholesterol to all formulations may have hindered encapsulation. This component prevents the phase transition of the lipid bilayers and always maintains them in a state of intermediate fluidity, tending to be more rigid, which hinders the incorporation of molecules (Roy et al., 2016).

**Table 5:** Efficiency of encapsulation for the formulations F1, F2, and F3.

Formulation	Extrusion (%)	Sonication (%)
F1	17.7	34.5
F2	9.5	12.2
F3	12.7	14.2

However, EE was higher for formulations produced by sonication, with the highest value of 34.5% for F1. This formulation also showed the highest efficiency among the formulations obtained by extrusion, with a value of 17.7%. F1 is composed of DPPC and cholesterol, DPPC has *T*_m_ (phase transition temperature) of 42°C, with a room temperature of 25°C bilayer is in its gel phase and rigid. Even so, it is the formulation that incorporated the largest amount of the peptide for the two techniques used.

The absence of other components contributed to the incorporation of the peptide according to this methodology. The presence of PEG in F2 and POPG in F3 made it difficult to incorporate 0WHst 5 into the liposomes. The encapsulation efficiency for F3 was little higher when compared to F2. The presence of POPG in this formulation aims to favor the crystalline liquid phase of the bilayer and grant more fluidity, since this lipid has *T*_m_ of −2°C, contributing the incorporation of the peptide. The lower EE was for F2, which has PEG in its composition. However, the presence of this component increases the physical-chemical stability of the liposomes, as will be seen in the following results. The increase in stability prolongs their presence in the human organism, by decreasing the uptake of the vesicles by the cells of the immune system and favors adherence in mucous membranes (Ron-Doitch et al., 2016; Jøraholmen et al., 2017), which may be a desirable characteristic for the intended application.

### Stability Studies

**Figure 2:**
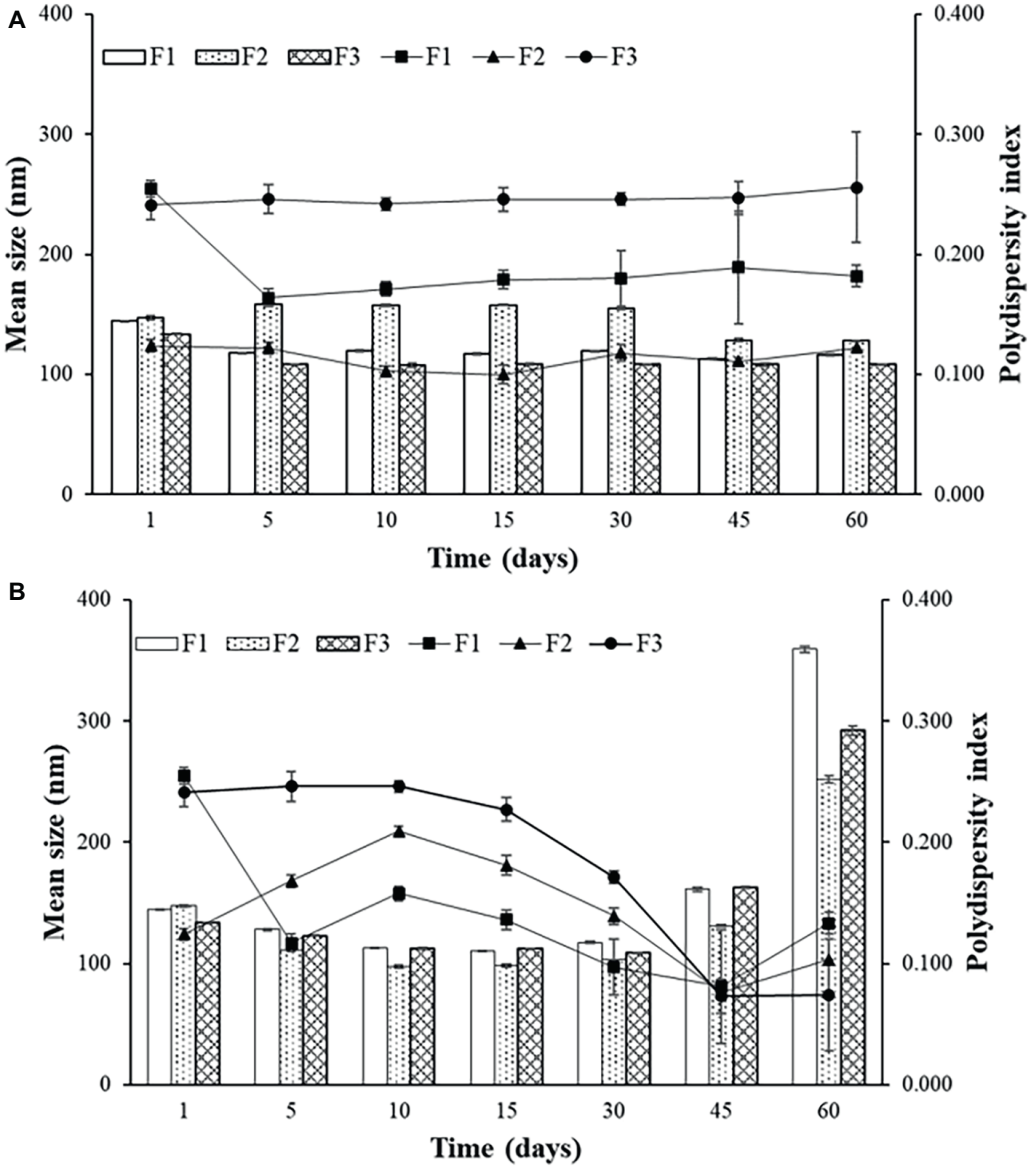
Mean size and polydispersity index (PDI) for F1, F2, and F3 obtained by sonication stored at 4°C **(A)** and at 37°C **(B)**.

According to Figure 2A, there were slight changes in average size and PDI for the sonicated F1 stored at 4°C. For this reason, sonication produced liposomes with better stability than the extrusion, maintaining the formulation stable during 60 days at 4°C. Storage at 37°C was more damaging to all formulations, providing many variations on mean size and PDI over time, which was also observed by Çelik et al. (2017).

The temperature of 4°C favors the storage of liposomes obtained by both techniques. It can be observed that for F2 (Figures 2A,B) up to 30 days, the average size and PDI did not show great variations, and after 45 days, a small variation of average size, from approximately 160 to 130 nm for sonication and from 160 to 110 nm for extrusion, with increase of PDI. Sonicated F1 and F2 presented better parameters and better stability when stored at 4°C. F2 has PEG in its composition, which according to some studies favors the maintenance of vesicle stability as also observed by Rai et al. (2008). Even though it does not favor encapsulation efficiency, as discussed previously, this component helps to maintain the stability of the formulation, which remains intact for longer when stored at 4°C.

**Figure 3:**
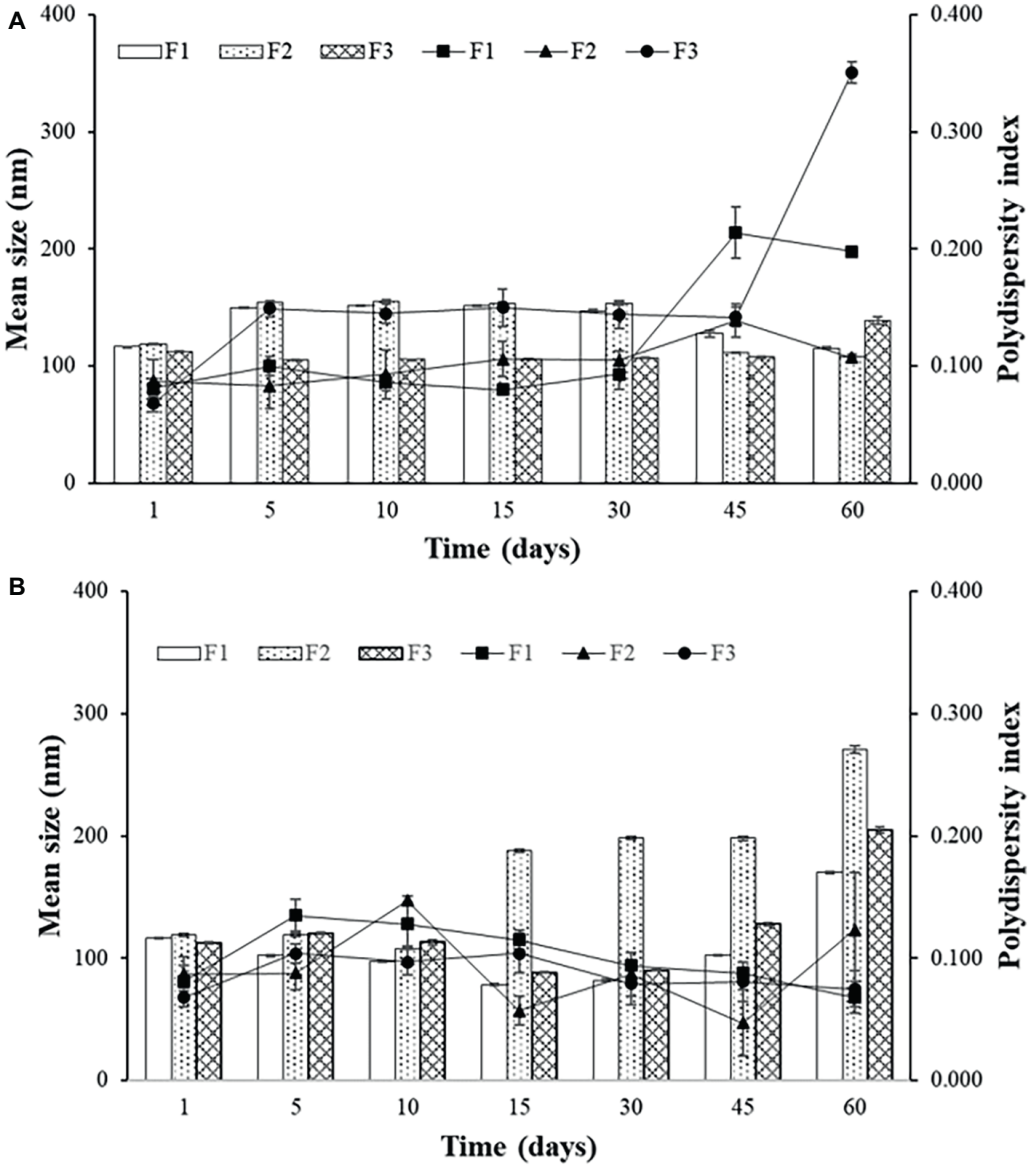
Mean size and polydispersity index (PDI) for F1, F2, and F3 obtained by extrusion stored at 4°C **(A)** and at 37°C **(B)**.

Sonicated F3 is the most stable formulation compared to F1 and F2, according to the graphs of Figures 2A and 3A.

In general, the best storage temperature is 4°C for all formulations. The technique that presents better stability is sonication. The lipids used in the liposomes may influence the stability, since the best stability was for F3, which has POPG in its composition. This component provides a more fluidity to the lipid bilayer, which can generate better adaptation throughout the storage period. POPG also confers a greater amount of negative charges to the bilayer, which difficult the aggregation of the liposomes. F2, which contains PEG, also showed better stability than F1, which has only DPPC and cholesterol. The addition of different lipids to the liposomes has made peptide encapsulation more difficult as previously seen, but may improve stability, as seen in these results.

### *In vitro* Release Study

**Figure 4:**
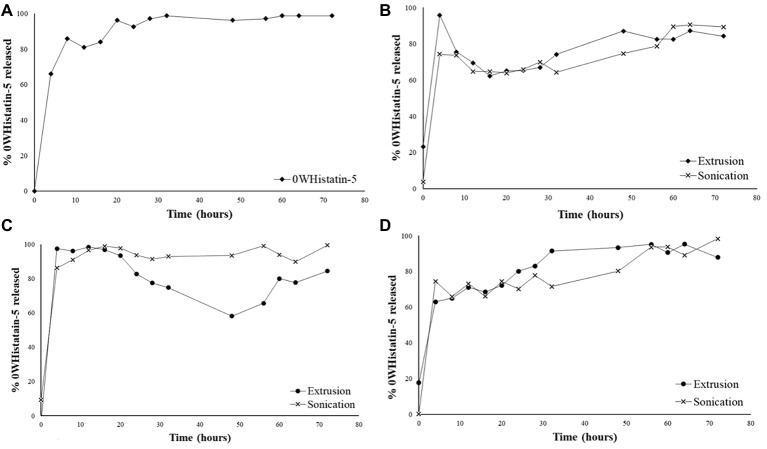
Release profile for: free 0WHistatin 5 **(A)**; F1 **(B)**; F2 **(C)**; and F3 **(D)**, loaded with 0WHistatin 5.

Graph A of Figure 4 represents the crossing of the unencapsulated peptide over the dialysis membrane. This test was performed as a control for the release experiments of the developed liposomal system. Thus, it was possible to know the time required for the non-encapsulated peptide to leave the dialysis membrane, once the peptide encapsulated in the liposomes would only come out after its rupture, which would take a longer time. The total crossing over of the unencapsulated peptide occurs after 5 h. For F1, F2, and F3 (Figures 4B–D), a 5-h time release peak is observed, similar to the unencapsulated peptide release profile.

The release profile indicates the occurrence of burst effect, which is characterized by the large amount of drug that is released in the first 24 h (Huang and Brazel, 2001). This effect is advantageous since the release of a large amount of 0WHst 5 in a short period of time can be used as the dose of attack to inhibit the growth of microorganisms moments after its application at the site of action. Because it is a release system that is intended for topical application for the treatment of oral lesions caused by *C. albicans*, a rapid release directly at the site of action would rapidly inhibit the proliferation of the microorganism (Huang and Brazel, 2001).

For all formulations, the profiles observed for extrusion and sonication were similar, and the use of distinct techniques for the production of liposomes does not interfere with their release kinetics. The burst effect observed in the first 5 h indicates that the released content is not encapsulated in the liposomes, or that it may be interacting with its outer surface, as also observed by Calienni et al. (2017). Thus, the release process for these liposomes occurs in two steps, initially there is a rapid release, followed by a drop indicating the dialysis system’s equilibrium due to osmotic forces. In a second moment, release of the remaining amount of the peptide occurs slowly for 80 h. The slow release step is related to disruption of the liposomes, which thereby releases the peptide contained therein (Lopes et al., 2012).

The release kinetics for nanocarriers is related to their encapsulation efficiency (EE) and stability. A higher EE will certainly result in a higher release peak, especially if burst effect occurs (Luan et al., 2006). Thus, in general, the formulations obtained by sonication, which have higher EE, also show greater increase in their second moment in the release profile.

### Killing Curve Studies

The experimental points shown in the graphs were calculated according to Equation 2, where *F* is the dilution factor, *C* is the colony forming unit count, and *V* is the volume of the portion used in the final dilution. The uncertainty of each experimental point was calculated from the standard uncertainty values and expanded uncertainty according to Corry et al. (2007) and Niemi and Niemelä (2001). The error bars do not appear in the graphs presented, since the expanded uncertainties presented results of one to two orders of magnitude lower than the experimental values.

$$y\left(UFC/ml\right)\;\;=\;\;F•\frac{C}{V}\;$$ (2)

ATCC 90028 was used in this test because it is considered a standard strain for screening tests for new antifungal agents. Besides, this study is based on the proposal of developing a liposomal system capable of prolonging the release of the anti-fungal peptide Histatin 5. The idea emerged from two studies of our research group involving this peptide for the treatment and prevention of oral candidiasis, mainly caused by *C. albicans* (Moffa et al., 2015a,b).

Previously, MIC assay was performed, and the values found for this strain were 128 μg ml^−1^ for fluconazole and 257.8 μg ml^−1^ for Histatin 5, as well as for the analog peptide 0WHistatin 5. The concentration of 512 μg ml^−1^ for fluconazole in the time kill assay represents four times the MIC value, since the intention was to visualize the cell death caused by the drug, which was used as a positive control. For the peptide, the MIC value was used since it was also the value used to prepare the liposomes and perform the encapsulation efficiency tests.

MIC assay was performed following the M27-A3 methodology of the Clinical and Laboratory Standards Institute Manual with the peptides 0WHistatin 5 and Histatin 5 using SB medium. This medium was chosen to provide enough nutrients to the microorganism during the 72 h of the time kill assay. It was necessary that there was enough amount of nutrients in the medium to promote the microorganism growth for the total time, because the developed system showed release for more than 60 h. Therefore, the observed death effect would be caused by the action of the peptide 0WHistatin 5 and not due to lack of nutrients.

The obtained result is consistent with the literature, and the MIC value for Histatin 5 and 0WHistatin 5 was 64.45 μg ml^−1^. Thus, the use of the SB medium did not interfere with the antifungal activity of the peptide and indicated that it can be used to perform time kill assays.

To determine MIC for the liposomal preparations is not possible, because the encapsulated peptide is in the liposome internal aqueous compartment, and, when serial dilutions are made, only the number of liposomes present in solution is diluted, while their internal concentration is not.

The internal concentration was determined by calculating the encapsulation efficiency. Therefore, performing a serial dilution test, such as the MIC, would not give us an enlightening result regarding the concentration of liposomal system to be used. Thus, it was determined 1 ml of liposomal system for use in the tests to make it possible to apply the value obtained in the encapsulation efficiency tests to estimate the concentration of the encapsulated peptide.

**Figure 5:**
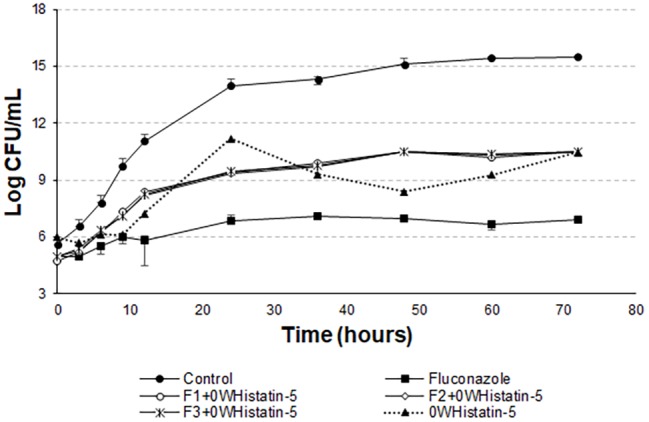
Time-kill curves for *C. albicans* (ATCC 90028) treated with extruded F1, F2, and F3 loaded with 0WHistatin 5. The lines referring to F1 + 0WHistatin 5, F2 + 0WHistatin 5, and F3 + 0WHistatin 5 systems are overlapping because the number of counted colonies were close.

Data of the time-kill assay (Figure 5) show that for 12 h the peptide inhibited the growth of the microorganism the same way that fluconazole. A dose of 257.8 μg ml^−1^ of 0WHst 5 and a dose of 512 μg ml^−1^ of fluconazole were used, demonstrating that 0WHst 5 in a lower dosage is capable of performing the same inhibition. This result is in agreement with Moffa et al. (2015b), which found inhibition in the range of 800–25 μg ml^−1^ for Hst 5. When incubating the peptide with *C. albicans* for only 1.5 h, Moffa et al. (2015b) obtained an approximate 2-log reduction in the CFU ml^−1^. As shown in the graph of Figure 5, after 12 h at 4-log reduction in CFU ml^−1^ was observed.

The *t*-student test showed that the means of the last point of the growth control curve and the last point of the curves F1 + 0WHistatin 5, F2 + 0WHistatin 5, F3 + 0WHistatin 5, and 0WHistatin 5 were statistically different, at the level of significance of 90%. This occurred for the systems produced by extrusion and sonication and showed that the produced liposomal system was effective in controlling the growth of *C. albicans*. This demonstrates that with prolonged treatment with 0WHst 5, it is possible to further reduce the growth of the microorganism.

**Figure 6:**
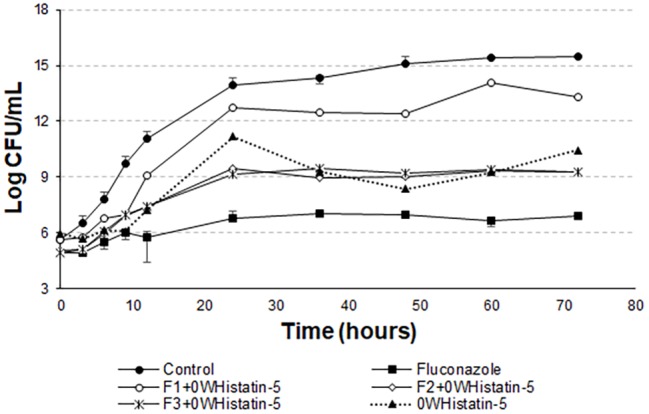
Time-kill curves for *C. albicans* (ATCC 90028) treated with sonicated F1, F2, and F3 loaded with 0WHistatin 5. The lines referring to F2 + 0WHistatin 5and F3 + 0WHistatin 5 systems are overlapping because the number of counted colonies was close.

However, Moffa et al. (2015a) proves that Hst 5 undergoes proteolytic action when present in human saliva, in addition to complexing with the enzyme salivary amylase, which leads to a decrease in the antifungal action of the peptide. These two effects occur rapidly as soon as the peptide is mixed with the total human saliva content. Encapsulating this peptide in liposomes is an attempt to protect it from these actions, allowing it to act for an extended period when added to its site of action. The data for the liposomal systems developed in this work (Figures 5 and 6) demonstrated prolonged activity for all the produced formulations, as well as the peptide 0WHistatin 5. This proves that the encapsulated peptide does not lose its action and maintains its antifungal potential. In addition, encapsulating the peptide in the liposomes improves the growth limitation, as it becomes more stable than for the non-encapsulated peptide. In Figure 5, after 24 h of incubation, there was a 3-log reduction in CFU ml^−1^, which remained constant during the 72-h test, reaching a reduction of 4 logs in CFU ml^−1^ at the end. For liposomes obtained by sonication (Figure 6), after 24 h incubation, the reduction was 4 logs for F2 and F3 and 2 logs for F1. This reduction was also maintained over the 72-h test period. For F2 and F3, the reduction was 6 logs at 72 h.

Han et al. (2016) inhibited close to 100% of *C. albicans* cells by treating them with 16 μg ml^−1^ Hst 5, after a total time of 1 h. This demonstrates that this peptide acts very well in the first few moments after addition to the medium. Thus, this study demonstrates that the use of a nanocarrier, such as liposomes, is able to prolong the time of peptide action, inhibiting the growth of the microorganism for 72 h.

The *t*-student test was applied to compare the last points of the F1, F2, and F3 curves with the last point of the growth control curve. For F1, the means compared are statistically equal. For F2 and F3, the means are statistically different. However, it can be noted from Figure 8 that there is no pronounced cell death as in Figures 5 and 6.

**Figure 7:**
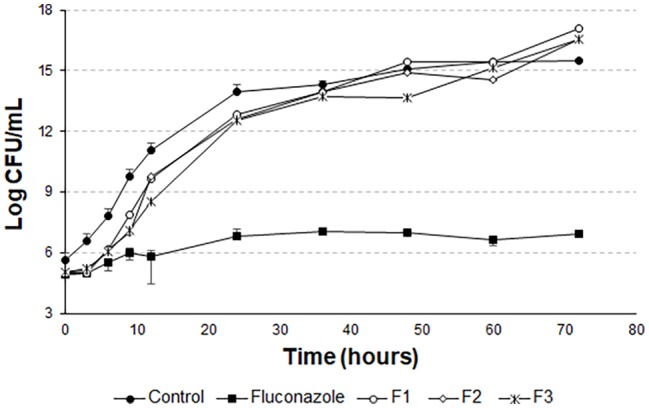
Time-kill curves for *C. albicans* (ATCC 90028) treated with sonicated and empty F1, F2, and F3.

**Figure 8:**
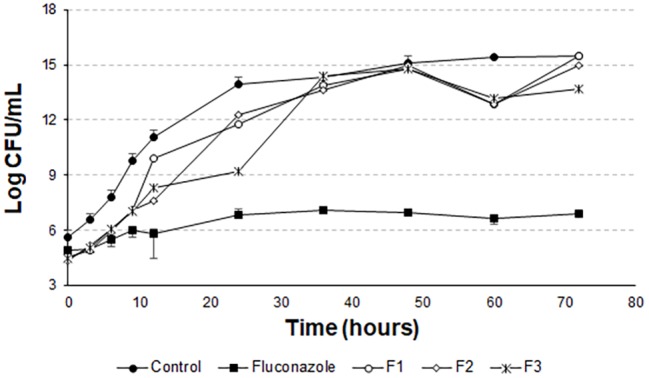
Time-kill curves for *C. albicans* (ATCC 90028) treated with extruded and empty F1, F2, and F3.

In Figure 7, the means of the last points of F1, F2, F3, and growth control are statistically different. There is also no pronounced cell death, as in Figure 8. This demonstrates that the liposomal system without the peptide 0WHst 5 does not affect the growth of *C. albicans*, with 0WHst 5 being responsible for the effect discussed above.

## Conclusion

The results obtained in this work indicate that the extruded liposomes are more homogeneous while the sonicated liposomes have a greater polydispersity. However, encapsulation efficiency values were higher for sonicated liposomes, which also produced more stable liposomes during storage at 4°C, among which F3 was the most stable. The results observed in the release kinetics studies indicate that there is a relationship between the stability of the liposome formulation and the release profile over time, the liposomes exhibit good stability, which controlled and prolonged release of the peptide. The time-kill results using ATCC 90028 showed that the liposomal systems preserved the antifungal activity of the peptide and were able to limit yeast growth for 72 h. As the results were promising and demonstrated to limit the growth of this microorganism, the intent of the research group is testing the systems for other strains, including clinical isolates and resistant strains of *C. albicans*. We can conclude that the liposomal system produced has the potential to limit the growth of the microorganism. Complementary tests, such as system degradation in human saliva and *in vivo* tests are the future steps off this work.

## Author Contributions

CZ performed all the experiments and wrote the manuscript. FF assisted in the time kill test and collaborated with the manuscript correction. EJ carried out the purification and characterization by mass spectroscopy of the peptide and critically reviewed the manuscript. PS characterized liposomes and assisted in the discussion of these results. FP elaborated, analyzed the data, and contributed to the discussion of time kill tests. MC and SG supervised the project and did the final revision of the manuscript.

### Conflict of Interest Statement

The authors declare that the research was conducted in the absence of any commercial or financial relationships that could be construed as a potential conflict of interest.
